# Use of plasma protein fraction in patients with septic shock admitted to the ICU

**DOI:** 10.1186/cc10382

**Published:** 2011-10-27

**Authors:** D Juneja, O Singh, P Nasa, Y Javeri, R Dang

**Affiliations:** 1 Department of Critical Care Medicine, Max Super Speciality Hospital, Saket, New Delhi, India

## Introduction

Certain colloids like albumin and plasma protein fraction (PPF) have been derived from human plasma and they are used as plasma expanders to treat patients with shock. PPF, which more closely resembles plasma in its constituents, contains albumin plus α and β globulins. We conducted this study to assess the effect of PPF on need for vasopressors, organ support and ICU mortality in patients with septic shock.

## Methods

A retrospective study was conducted and data were collected from the records of patients admitted to a 16-bed neuro and medical ICU over a 1.5-year period. All adult patients admitted with septic shock and requiring vasopressor support (for more than 6 hours) in spite of aggressive fluid resuscitation were enrolled. Patients who were transferred from some other ICU or ward and those who developed shock during their ICU course were excluded from the analysis. Patients were divided into two groups: patients in whom PPF was used along with resuscitative fluids comprised the study group, whereas others formed the control group. Patients in these groups were compared according to need for organ support, ICU mortality and time taken to stop vasopressor agents. PPF (Plasmanate^®^) was administered in a protocolized way at the dosage of 10 to 20 ml/hour for the first 48 hours. Development of any complication like allergy or hypotension associated with PPF was also noted.

## Results

There was no significant difference in the baseline characteristics of patients in both groups in terms of age (*P *= 0.154), sex (*P *= 0.479), severity of illness (APACHE II score, *P *= 0.356), and presence of organ failure (SOFA score, *P *= 0.105). Among the outcome parameters there was no significant difference in terms of need for renal support (*P *= 0.814), mechanical ventilation (*P *= 0.776), ICU stay (*P *= 0.122), hospital stay (*P *= 0.054) and ICU mortality (*P *= 0.091). However, there was a significant difference in time taken to stop the vasopressors (*P *= 0.030) (Table 1). There were no incidences of any complications or side effects in any group.

**Table 1:** Comparison between patient characteristics and ICU course among patients in control and PPF groups

Parameter of interest	Control group (*n *= 87)	PPF group (*n *= 99)	*P *value
Age (years)	64.11 ± 15.4	67.28 ± 14.8	0.154
Sex, male	50 (57.5%)	63 (63.6%)	0.479
APACHE II score	20.69 ± 6.2	21.64 ± 7.6	0.356
PDR	39.51 ± 19.2	42.47 ± 22.5	0.341
SOFA score	9.53 ± 3.5	10.38 ± 3.7	0.105
RBC transfusions	32 (36.8%)	33 (33.3%)	0.735
Renal support	31 (35.6%)	38 (38.4%)	0.814
Mechanical ventilation	50 (57.5%)	60 (60.1%)	0.776
Time taken to stop vasopressors (hours)	**70.69 ± 55.2**	**51.29 ± 64.5**	0.030*
ICU stay (days)	10.38 ± 11.4	13.42 ± 14.8	0.122
Hospital stay (days)	12.91 ± 11.8	16.83 ± 15.3	0.054
ICU mortality	34 (39.1%)	52 (52.5%)	0.091

*P < 0.05. Bold text indicates statistically significant.

## Conclusion

PPF may be used safely and effectively for initial resuscitation of patients with septic shock requiring vasopressor support. It may lead to early termination of vasopressor support; however, it did not translate to lesser need for organ support or reduced ICU mortality in our patient cohort. To demonstrate such benefits, larger multicenter trials are warranted.

